# Knowledge Gaps and Current Evidence Regarding Breastfeeding Issues in Mothers with Chronic Diseases

**DOI:** 10.3390/nu15132822

**Published:** 2023-06-21

**Authors:** Rozeta Sokou, Stavroula Parastatidou, Zoi Iliodromiti, Katerina Lampropoulou, Dionysios Vrachnis, Theodora Boutsikou, Aikaterini Konstantinidi, Nicoletta Iacovidou

**Affiliations:** 1 Neonatal Intensive Care Unit, “Agios Panteleimon” General Hospital of Nikea, 3 D.Mantouvalou Str., Nikea, 18454 Piraeus, Greece; kmaronia@gmail.com; 2 Neonatal Department, School of Medicine, National and Kapodistrian University of Athens, Aretaieio Hospital, 11528 Athens, Greece; theobtsk@gmail.com (T.B.); niciac58@gmail.com (N.I.); 3 Neonatal Intensive Care Unit, “Elena Venizelou” Maternity Hospital, 11521 Athens, Greece; stavroula.parastatidou@gmail.com; 4 Neonatal Intensive Care Unit, School of Medicine, University of Ioannina, 45110 Ioannina, Greece; katerina.lambropoulou@yahoo.gr; 5 Endocrinology Unit, 2nd Department of Obstetrics and Gynecology, School of Medicine, National and Kapodistrian University of Athens, Aretaieio Hospital, 11528 Athens, Greece; dionisisvrachnis@gmail.com

**Keywords:** breastfeeding, maternal chronic disease, neonates, perinatal outcomes

## Abstract

The prevalence of chronic maternal disease is rising in the last decades in the developed world. Recent evidence indicated that the incidence of chronic maternal disease ranges from 10 to 30% of pregnancies worldwide. Several epidemiological studies in mothers with chronic diseases have mainly focused on the risk for adverse obstetric outcomes. Evidence from these studies supports a correlation between maternal chronic conditions and adverse perinatal outcomes, including increased risk for preeclampsia, cesarean section, preterm birth, and admission in the Neonatal Intensive Care Unit (NICU). However, there is a knowledge gap pertaining to the management of these women during lactation. This review aimed at summarizing the available research literature regarding breastfeeding in mothers with chronic diseases. Adjusted and evidence-based support may be required to promote breastfeeding in women with chronic diseases; however, our comprehension of breastfeeding in this subpopulation is still unclear. The literature related to breastfeeding extends in various scientific areas and multidisciplinary effort is necessary to compile an overview of current evidence and knowledge regarding breastfeeding issues in mothers with chronic diseases.

## 1. Introduction

Breastfeeding is the best and most natural nutrition for infants. Through breastfeeding, infants are offered all the necessary nutrients and elements for their optimal growth and development. The World Health Organization, Unicef, and the American Academy of Pediatrics recommend exclusive breastfeeding for the first 6 months of life and continuation of breastfeeding (after introduction of solid foods at 6 months) until the first year of life and for as long as the mother and child desire [1,2,3]. There is indisputable evidence in the literature regarding breastfeeding benefits for the infant, the mother, the family, and the society, in general [4]. Maternal milk contains the ideal qualitative and quantitative composition for optimal neonatal growth. Breastfeeding contributes to the smooth physical and psychological development of the infant, conferring short-term as well as long-term benefits. First of all, breastfed infants have a decreased risk of childhood mortality [5,6,7]. Research has revealed that infants who have been breastfed for less than two months and those who are partially or not breastfed have a higher mortality risk compared to exclusively breastfed infants [8,9]. Breastfeeding for more than six months protects against obesity, diabetes, asthma, cardiac conditions, and increases final height [10,11,12,13,14]. Rich-Edwards et al. [14] investigated the association between breastfeeding and cardiac conditions and suggested that breastfed infants may present a lower risk of ischemic cardiovascular disease in adulthood. Additionally, breastfed children seem to have lower risk of developing certain types of childhood cancer, including leukemia and lymphomas [15,16]. Breastfeeding positively impacts cognitive, emotional, and social development of the infant [17,18]. Neonatal mortality and morbidity is reduced in breastfed neonates, in particular preterm newborns. Breastfeeding fortifies the immune system, promoting immune maturation and protecting infants against infections. Breastmilk interacts with gut microbiota and, to a degree, shapes microbiome colonization, with possible effects on long-term programming [19,20].

Breastfeeding contributes to the regular physical and psychological development of the infant, with short-term and long-term advantages. The majority of mothers are able to breastfeed and entitled to it, providing they are offered accurate information and are supported by family, healthcare system, and society.

The presence of a chronic disease is increasingly common in pregnant women, with a frequency of up to 10–30% [21,22]. The prevalence of chronic maternal disease is rising in the last decades in the developed world, with a reported increase from 4% in 1989 to 16% in 2013 [21]. This trend is possibly explained by the rise in disease rates in the general population, the increase in mean childbearing age of women, and the medical progress in assisted reproduction. Research in mothers with chronic diseases has mainly focused on their risk for adverse obstetric outcomes. Evidence from these studies supports a correlation between maternal chronic conditions and adverse perinatal outcomes, including increased risk for preeclampsia, cesarean section, preterm birth, and admission in the Neonatal Intensive Care Unit (NICU) [23,24,25,26,27]. However, there is a knowledge gap pertaining to the management of these women during lactation. The aim of this review was to summarize the available literature regarding breastfeeding in mothers with chronic diseases. Data from published studies are analyzed below and summarized in a relevant table in Supplementary Materials (Table S1).

## 2. Autoimmune Diseases and Breastfeeding

**Figure 1:**
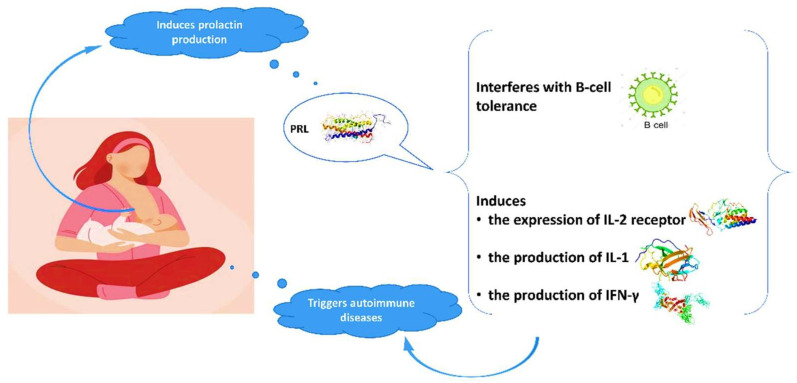
Interaction of prolactin with autoimmune system.

Advantages of breastfeeding in mothers with autoimmune diseases are outlined in the literature. The high prevalence of autoimmune conditions among women indicates the crucial role of gender and hormonal implication in the pathogenesis of these diseases. Evidence suggests a relationship between prolactin and autoimmune diseases, in particular systematic lupus erythematosus (SLE), rheumatoid arthritis (RA), and peripartum cardiomyopathy (PPCM) [28,29,30,31,32,33]. Prolactin is mainly produced in the pituitary gland and its role is not only to stimulate the growth of the mammary gland and the production of milk during lactation, but also to modify the maternal behavior. Genes coding for prolactin are located in chromosome 6, close to HLA-DRB1. Polymorphisms of the human prolactin gene may affect the pathogenesis of autoimmune conditions [29,34]. Elevated prolactin levels may interfere with B-cell tolerance through various mechanisms [28,35], while prolactin induces the expression of IL-2 receptor and the production of IFN-γ and IL-1 (Figure 1). It modifies the maturation of dendritic and thymus cells, leading to IFN-α production and enhancement of pro-B-cells generation.

### 2.1. Systematic Lupus Erythematosus and Breastfeeding

Systematic lupus erythematosus (SLE) is a chronic, multisystemic, inflammatory disease of autoimmune etiology, commonly presented in young women of reproductive age. Prolactin has been found to be implicated in the pathogenesis of antiphospholipid syndrome and the observed impaired fertility [36,37,38]. Recently, Song et al. [31], in a meta-analysis, revealed a significantly positive correlation between prolactin and SLE activity. In a large, cohort study by Orbach et al. [39], SLE patients with hyperprolactinemia presented with significantly more episodes of pleuritis, pericarditis, and peritonitis, and had more frequently anemia and proteinuria compared to patients with normal prolactin levels. The authors concluded that dopamine agonists could be a potential treatment for SLE patients with hyperprolactinemia. In other studies, treatment with bromocriptine has reduced disease activity, and therapy cessation was associated with SLE flares [36,37]. Current evidence supports the benefits of treatment with bromocriptine in refractory SLE or in the prevention of flares after labor [37]. Conclusively, these findings question whether mothers with SLE should breastfeed.

Contrary to the numerous prospective and retrospective studies published on pregnancy outcomes of women with SLE [40,41], data regarding breastfeeding are currently scarce. Breastfeeding rates and duration seem to be decreased in SLE patients. Orefice et al. [42] in a cohort study reported that the vast majority of mothers with SLE (96.5%) were planning to breastfeed during pregnancy and 71.9% did breastfeed. However, half of these patients ceased breastfeeding after 3 months. Additionally, factors including cesarean section, preterm birth, intrauterine growth restriction (IUGR), and disease flares were positively correlated with non-breastfeeding. Also, a relationship between treatment with hydroxychloroquine (HCQ) and delayed breastfeeding cessation was reported for the first time. In subsequent studies, HCQ has shown reduction in disease relapse risk during and after pregnancy [43,44], decreased rates of recurrent neonatal lupus and improvement of labor outcome [45,46]. In another study, Noviani et al. reported that 49% of participating SLE patients decided to breastfeed [47]. This rate was not significantly affected by socioeconomic factors. Furthermore, disease activity after labor, full-term labor, breastfeeding education and planning were positively correlated with breastfeeding. Transfer of HCQ, azathioprine, methotrexate, and prednisone to maternal milk seems very limited and all drugs are compatible with breastfeeding [47]. Acevedo et al. [48] recorded reduced breastfeeding rates and duration in SLE patients: they breastfed their children half of the time that the mothers without SLE did (6 months vs. 12 months, respectively). The initiation of a new treatment was the main reason for breastfeeding cessation in spite of the fact that these drugs were low risk for breastfeeding. Breastfeeding duration could be improved by enhancing the level of information provided to patients. Complications during the postnatal period were mainly responsible for not initiation of breastfeeding. HCQ is compatible with breastfeeding according to the American Academy of Pediatrics (AAP), and most SLE specialists recommend continuation of breastfeeding in SLE patients receiving antimalarial medicines [49,50]. Prednisone and ibuprofen in low doses are also acceptable options during pregnancy, while data on the use of other medicines for the treatment of SLE in pregnancy and lactation are limited. Current recommendations advocate for the initiation of HCQ when pregnancy is scheduled and the continuation of the drug throughout pregnancy and lactation [51].

### 2.2. Rheumatoid Arthritis and Breastfeeding

High prolactin levels may result either in autoimmune disease presentation in mothers with predisposition or in flares in patients with existing conditions [52]. Risk for RA onset increases during the postpartum period, particularly after the first pregnancy [53]. Women who developed RA within 12 months of the first pregnancy were five times more likely to have breastfed, while breastfeeding rates sharply declined in a subsequent pregnancy [54]. Barrett et al. [55] compared disease activity during and for 6 months after pregnancy between 49 patients who did not breastfeed, 38 who breastfed for the first time and 50 who had previous breastfeeding experience. Following adjustment for possible confounding factor, including treatment, patients who were breastfeeding for the first time showed increased disease activity 6 months after labor, indicated by self-reported symptoms, number of affected joints, and C-reactive protein levels, suggesting that this flare could be caused by breastfeeding. Brennan and Silman [54] investigated whether the presentation of RA after labor could be attributed to breastfeeding. Through a media campaign, the authors interviewed 187 women who presented RA within 12 months of labor and compared their breastfeeding history with that of 149 women of similar age selected from the patient registers of a nationwide group of general practitioners. In total, 88 women developed RA after their first pregnancy, and 80% of them breastfed. This rate was higher than the prevalence of breastfeeding (50%) in the 129 controls (adjusted odds ratio (aOR) 5.4, 95% confidence intervals (CI) 2.5–11.4). The risk for RA development was less increased following breastfeeding in a second (OR 2.0) and not increased in a third pregnancy (OR 0.6). More recently, Eudy et al. [56] in a cross-sectional study reported that most women with RA breastfed and were regularly receiving treatment during lactation. Disease activity seemed to worsen, in particular for the patients who did not receive treatment during lactation, while improvement was only observed in women who followed treatment during breastfeeding. Ince-Askan et al. [57] in a prospective cohort study concluded that only 4% of mothers with arthritis exclusively breastfed until 26 weeks compared to 25% of the general population.

### 2.3. Idiopathic Inflammatory Bowel Diseases and Breastfeeding

Idiopathic inflammatory bowel diseases (IBDs) are chronic intestinal disorders usually diagnosed during the second and third decades of life. The effect of pregnancy on the course of disease varies; the majority of patients remain in remission, while a few of them improve probably due to generalized immunosuppression during gestation. Contrarily, 1/3 of patients deteriorate [58,59]. Although the exact mechanisms explaining aggravation in pregnancy are not known, it is speculated that the cause may be the lack of maternal- fetal immunocompatibility [60]. Consequently, women with IBD may present with disease activation and need treatment at conception, pregnancy, or labor. Women with IBD are at increased risk for spontaneous abortions, preterm labor, and low birth weight neonates [59,61]. Some researchers suggest an increased risk of chromosomal disorders (although the role of disease activity relative to that of the drug exposures has not been elucidated) and adverse perinatal outcomes in patients with IBD [59,62]. Breastfeeding rates in women with IBD range among studies. Dotan et al. [62] reported that mothers with IBD breastfed less frequently. Approximately 1/3 of them did not breastfeed at all compared to 1/5 of healthy controls (*p* < 0.005), and short-term and long-term breastfeeding were also less common in mothers with IBD. Moreover, mothers who received treatment with immunomodulators and steroids had significantly lower breastfeeding rates in comparison to women who were only administered 5-ASA. In a study by Moffatt et al. [63], 83.3% of IBD patients began to breastfeed compared to 77.1% of the general population (*p* > 0.05). With regards to breastfeeding duration, 56.1% of IBD patients vs. 44.4% of the general population breastfed for more than 24 weeks (*p* = 0.02) [63]. The rate of disease flare during the first year after labor was 26% for breastfeeding and 29.4% for non-breastfeeding patients with Chron’s disease (CD, *p* = 0.76) and 29.2% for breastfeeding vs. 44.4% (*p* = 0.44) for non-breastfeeding women with ulcerative colitis (UC). The risk for disease flare was independent of age, disease length, and socio-economic status. The authors concluded that IBD does not seem to reduce chances of breastfeeding. Lactation is not associated with increased risk of flares; contrarily, it could be protective during the first year after labor. Kane et al. [64] studied 122 women with IBD who were asymptomatic during pregnancy. Only 44% breastfed due to doctor recommendations, fear of drug interactions, and personal choice. Of those who breastfed, 43% presented postpartum disease flare. Non-adjusted OR for disease activity in women with breastfeeding history was 2.2 (95% CI 1.2–3.9, *p* = 0.004). After risk stratification by disease type, OR for UC was 0.89 (95% CI 0.29–2.7, *p* > 0.05), and for CD it was 3.8 (95% CI 1.9–7.4, *p* < 0.05). Following adjustment for treatment cessation, statistical significance of OR was not retained. These results indicate that a significant number of IBD patients do not breastfeed. A relationship between breastfeeding and disease activity may be owing to IBD treatment cessation. 

Breastfeeding has been associated with prevention of IBD in children [62,65,66], a benefit which should be taken into serious consideration by mothers with IBD. 

### 2.4. Multiple Sclerosis and Breastfeeding

Multiple sclerosis (MS) is an autoimmune inflammatory disease in which sclerotic plaques are formed in the central nervous system causing neuronal demyelination and damage. MS is usually encountered in women of childbearing age [67], and although disease modifying treatments (DMTs) reduce relapse rates, none of these treatments are recommended during pregnancy or breastfeeding [68,69,70]. 

MS relapse rates are decreased during the last trimester of pregnancy, but they rise during the first 3 months after labor, with up to 30% of patients relapsing [71]. Postpartum relapses are associated with high risk of disability [71] and deterioration of existing disability [72]. Women are frequently faced with the dilemma of breastfeeding or not breastfeeding and re-initiating DMT after labor. Despite the many observational studies, there is no consensus to date as to whether there is a relationship between breastfeeding and postpartum relapse control [72,73,74,75]. In 2012, a meta-analysis showed that non-breastfeeding women had double the risk of postpartum relapse than breastfeeding mothers [76]. However, there is great heterogeneity among studies included in this meta-analysis, and researchers did not assess whether disease activity before pregnancy affected the findings of the study or if the results were attributed to exclusive breastfeeding and its different hormonal impact from non-exclusive breastfeeding. Krysko et al. [70], in a 2021 systematic review and meta-analysis, demonstrated that breastfeeding was correlated with lower rate of postpartum MS relapses, with this beneficial effect being greater in cases of increased disease activity and exclusive breastfeeding. For a mother with MS, and possibly mobility problems, breastfeeding advantages may help improve her quality of life and health. 

## 3. Epilepsy and Breastfeeding

Women with epilepsy often face unique problems and practical issues with pregnancy and lactation, and their breastfeeding rate tends to be low [77,78,79]. The main concern seems to be the possible risk to the child from the transfer of antiepileptic drugs (AED) in the maternal milk [80]. The 2009 guidelines on the management of pregnant women with epilepsy, issued by the American Academy of Neurology, highlight the lack of evidence regarding breastfeeding safety, which is stressful for both mothers and healthcare providers [81]. Reports of possible adverse events of AED, including suppression, irritability, hepatic dysfunction, or rash, in exposed infants are rare [82,83]. However, pharmacokinetic assessments are complex and published studies investigating AED concentrations in maternal milk and/or infant plasma are scarce [82]. Concerns have also been raised that the prolonged exposure to AED through breastfeeding might affect the developing brain, resulting in behavioral or mental disorders. Nevertheless, data from the Neurodevelopmental Effects of Antiepileptic Drugs (NEAD) indicated that breastfed children whose mothers received lamotrigine, carbamazepine, valproic acid, or phenytoin during pregnancy had higher intelligence quotient at 6 years compared to non-breastfed children [84]. Additionally, some women with epilepsy fear that breastfeeding increases sleep deprivation and possibly increases the risk of seizure episodes [79]. Studies have exhibited that mothers who breastfeed during the first 3 months tend to sleep more and less disturbed at night compared to non-breastfeeding mothers [85]. 

It seems that AED can be found in maternal milk; however, this exposure is limited compared to that of the transfer through placenta during pregnancy. Most AED are considered safe during lactation, and mothers with epilepsy should be encouraged to breastfeed [79]. Despite the recommendations in the literature, breastfeeding rates in mothers with epilepsy remain low [78]. 

## 4. Asthma and Breastfeeding

Asthma is the most common, severe, chronic respiratory disease, complicating 4% to 8% of pregnancies [86]. Approximately 1/3 of pregnant women suffer from asthma exacerbation during pregnancy, 1/3 remain stable, and 1/3 experience improvement [87]. This disease course variability is still under investigation [86,88].

There is evidence that asthma can negatively affect pregnancy outcome and that pregnancy may modify the clinical status of an asthmatic woman. Inadequately treated asthma may cause maternal hypoxemia, which can complicate pregnancy and labor outcome. Women with asthma are at increased risk of preterm labor, cesarean section, premature rupture of membranes, chorioamnionitis, placenta abruption, low birth weight neonate (the more compromised the pulmonary function, the lower the birth weight), extended hospital stay, and perinatal mortality [86,87]. The impact of breastfeeding in children of asthmatic mothers is unclear; some studies report a protective role against childhood asthma, while others report ambiguous results [89,90,91]. Meghan et al. [92], using data of 2773 infants from the Canadian Healthy Infant Longitudinal Development (CHILD), showed an inverse correlation between breastfeeding of asthmatic mothers and infant wheezing, independent of maternal smoking, education, and other risk factors (adjusted relative risk (aRR) 0.52, 95% CI 0.35–0.77 for ≥12 vs. <6 months of breastfeeding). Compared to non-breastfeeding, wheezing was reduced by 62% with exclusive breastfeeding, and by 37% with breastfeeding supplemented with solid foods at 6 months. Breastfeeding was not protective if supplemented with formula. Harvey et al. [89] studied the prevalence of wheezing, as reported by parents at 6 and 12 months of life. Breastfeeding for more than 6 months was associated with lower aRR at 6 months compared to no breastfeeding (aRR 0.54, 95% CI 0.30–0.96). Breastfeeding for more than 6 months was associated with lower risk for bronchiolitis and healthcare utilization in high-risk infants due to maternal asthma, both at 6 and at 12 months. The relationship between breastfeeding and asthma or recurrent wheezing varied depending on the age of the child, as well as the presence of maternal asthma or atopia in the child. In a study of 1246 children by Wright et al. [90], breastfeeding was correlated with a lower risk of recurrent wheezing during the first 2 years of life, but a higher risk of asthma and recurrent wheezing after the age of 6 years only for atopic children of asthmatic mothers.

Differences in concentrations and activation of leukocytes and cytokines have been described in the milk of mothers with and without asthma, differences which may alter the immunologic response of the infant. Although the specific mechanisms of immunologic alterations are yet to be elucidated, the relevant differences in the content of maternal milk may help explain the beneficial role of breastfeeding in children of asthmatic mothers [93].

Data demonstrate a lower breastfeeding initiation and duration and decreased rates of exclusive breastfeeding in women with asthma compared to those in the general population [94]. Breastfeeding rates tend to be lower in case of drug-treated asthma during pregnancy or postpartum [95]. 

Women with asthma should be assured that medical treatment is less dangerous for the fetus/infant than a severe exacerbation. Breastfeeding should continue in women receiving treatment, as only traces of the drugs are excreted in maternal milk. The decision to change a successful therapeutic regimen must be weighed against possible adverse effects of the drug continuation on the infant [96]. Breastfeeding does not impact the status of maternal asthma and, if appropriately controlled, asthma does not affect the duration of breastfeeding. Mothers should be encouraged to maintain a breastmilk stash that could be used in case of hospitalization or severe exacerbation which would obstruct breastfeeding.

## 5. Congenital Heart Disease (CHD) and Breastfeeding

Pregnancy is complicated by maternal CHD in 1–4% of cases. Data regarding worldwide prevalence of CHR in pregnancy are limited [97]. Sudden Adult Death Syndrome, PPCM, aortic dissection and myocardial infraction (MI) were the most common causes of maternal death in the UK during 2006–2008 [98,99]. The awareness of risk related to cardiovascular disease during gestation and the management of women with severe pre-existing cardiac conditions are vital for scheduling of pregnancy and monitoring for probable fetal and maternal morbidity and mortality [97]. Every treatment during pregnancy affects the mother as well as the fetus, and therefore the optimal management for both should be targeted. Scientific societies, including the American Heart Association (AHA), the American College of Cardiology (ACC), and the European Society of Cardiology (ESC), have published guidelines on the management of cardiac conditions during pregnancy [97]. Since there is lack of prospective or randomized clinical trials, recommendations and guidelines mainly correspond to level of evidence C [97]. Large registries and prospective trials are necessary for the enhancement of current knowledge. The ESC Registry of Pregnancy and Cardiac disease (ROPAC) and the EUROmediCAT registry of major congenital anomalies provide data on the epidemiology and exposure to medicines in pregnancy [100,101]. Due to progress in the diagnosis and surgical management of patients with CHD, the majority of these children grow up, and the number of pregnancies with underlying CHD is rising [102].

Hormonal changes cause significant hemodynamic alterations from the beginning of pregnancy, with increase in blood volume, heart rate, stroke volume, and cardiac output, and decrease in vascular resistance [103]. After labor, maternal blood volume decreases to gradually return to pre-gestation levels at 6 months postpartum. These hemodynamic adaptations may lead to cardiovascular events, including cardiac failure and arrythmias, in women with CHD [104]. 

Breastfeeding can also affect hemodynamic changes after labor; however, data on its impact on cardiac output or clinical course of patients with CHD are scarce. A study in rats exhibited significant increase in cardiac output in breastfed vs. non-breastfed animals [105]. Similarly, circulating blood volume and cardiac output were found increased during lactation in rabbits [106]. Lactating hormones, like prolactin and oxytocin, have been associated with aggravation of maternal cardiac diseases, including PPCM and aortic dissection in the context of Marfan syndrome [32,107]. Matsuzaka et al. [108] reported that women with CHD tend to opt for formula feeding from the first month postpartum. However, significant differences in postpartum cardiovascular events and levels of brain natriuretic peptide (BNP) associated with breastfeeding were not observed. 

ESC does not recommend breastfeeding for women with severe cardiac disease (class of recommendation IIb). If a joint decision is made on continuation of breastfeeding (in patients with mild to moderate cardiac disease), watchful use of medicines and assessment of probable adverse effects on the infant are recommended [97]. 

Breastfeeding does not increase arterial blood pressure. Anti-hypertensive agents are excreted in breast milk, usually in low concentrations, with the exception of propranolol and nifedipine, the levels of which are similar to maternal plasma [97,109].

## 6. Sickle Cell Disease and Breastfeeding

Sickle cell disease (SCD) in pregnancy is associated with a sixfold increase in maternal mortality and increased risk of gestational hypertension, birth of a small for gestational age (SGA) neonate, preterm birth, and stillbirth [110]. The risk for obstetric complications and perinatal mortality is higher in pregnant women with SCD [111]. Complications related to the disease, including painful crises prepartum and postpartum, pulmonary complications, anemia, preeclampsia, and eclampsia, are also higher in women with SCD [112]. In developed countries, pregnancies with SCD are actively monitored and managed, with improved results [110]. In the United States, pregnancy-related complications have declined during the last decades, despite the still elevated maternal mortality and the occurrence of spontaneous abortions and perinatal deaths [112]. 

There is no evidence that lactation accelerates painful crises and patients with SCD should be encouraged to breastfeed [113]. Hydroxyurea is the licensed treatment for the management of SCD in adults and children over 9 months [114]. As an inhibitor of ribonucleotide reductase, it has a potential teratogen and mutational effect, and its use in pregnancy and lactation is contraindicated. Nevertheless, women with SCD have an increased risk of morbidity and mortality during pregnancy and after labor; therefore, cessation or modification of the optimal treatment could be harmful for both mothers and infants. In women who breastfeed every 2–3 h, levels of hydroxyurea in the milk are 3.4% of the relative infant dosage, which is below recommended safety levels. Also, if mothers with SCD pump and discard the milk after administration of hydroxyurea, the percentage of the drug transferred through breastmilk is further reduced by 50%. In this case, the infant will be exposed to a drug dose of <1 mg/kg/day, much lower than the doses of 20–30 mg/kg/day used for the treatment of infants with SCD [114,115]. Therefore, and due to the minimal amount of hydroxyurea transferred through human milk, it is safe for lactating mothers to receive hydroxyurea [114]. Patients with SCD may need opioid analgesics for effective pain management during pregnancy and lactation. Neonates of mothers on chronic analgesic treatment should be monitored for symptoms of abstinence syndrome [113]. In addition, as opioids are excreted in breastmilk, decision on their long-term administration must be individualized [113]. 

## 7. Thalassemia and Breastfeeding

Current therapeutic management of patients with thalassemia has improved their prognosis, survival and quality of life. Thus, pregnancies in women with thalassemia are increasing, and awareness of the specific risk factors is vital for the proper monitoring and management of these patients and their fetuses. Pregnancy with thalassemia is considered to be high risk, and follow-up by a team of specialists is required. 

Low rates of breastfeeding in women with thalassemia could be explained by the necessity of re-initiation of treatments like chelates that are contraindicated during lactation [116,117]. Women with thalassemia who plan to breastfeed should begin deferoxamine (immediately after the 24 h infusion of deferoxamine postpartum). Deferoxamine is excreted in breastmilk but is not harmful for the neonate as it is not absorbed orally. Data on the safety of breastfeeding with other chelates are scarce [117].

## 8. Malignancies and Breastfeeding

Preservation of fertility following cancer has become feasible. Breastfeeding is also possible. In case of treatment, advantages of the drug over benefits of breastfeeding for the mother and the infant should be considered.

Reliable evidence regarding breastfeeding in women with a history of breast cancer is currently unavailable. Guidelines by the Society of Obstetricians and Gynecologists of Canada (SOGC) state that women should be encouraged to breastfeed since there is no evidence that breastfeeding increases the risk of relapse or development of a novel tumor or that it endangers infant health [118]. According to a study published in *The Lancet*, the total incidence of breast cancer in developed countries could be reduced by half (2.7 from 6.3 per 100 women up to 70 years old) if women had the mean number of births and duration of breastfeeding common in developing countries [119,120]. Breastfeeding represents almost 2/3 of the estimated decrease in breast cancer prevalence [118]. The observed protection is related to hormonal changes during lactation, which delay menstruation and reduce exposure to estrogens that are associated with risk of breast cancer [121]. Additionally, during pregnancy and lactation, apoptosis of breast cells is frequent, helping remove cells with possible DNA defects and decreasing risk of breast cancer [121,122].

However, a recent systematic review by Bhurosy et al. [123] reported that breastfeeding is challenging among breast cancer survivors. Conservative surgical and irradiation therapy may reduce but not eliminate the ability of the affected breast to produce milk. Other issues faced by cancer survivors include uncertainty concerning breastfeeding, lack of support by doctors and family members, lack of access to a certified lactation consultant (IBCLS), nipple pain and discomfort. Social and clinical factors associated with successful breastfeeding include breastfeeding motivation, consultation and support by a multidisciplinary team of healthcare professionals, family members, or friends, the use of the other breast, and lactation enhancement with appropriate diet and galactagogues [123].

Patients with chronic myelogenous leukemia (CML) who achieve optimal response with tyrosine kinase inhibitors (TKIs) have high survival expectancy and concerns are raised regarding family planning. TKIs are potentially teratogenic, classified as pregnancy category D by the FDA, and their use in pregnancy is not recommended unless the treatment benefits outweigh the possible risks [124]. The suggestion for TKI cessation and breastfeeding for a short period of 2–5 days after labor is also acceptable [124,125].

Reduction in risk for breast and ovarian cancer is among the benefits of breastfeeding. Specialists who follow-up pregnant and lactating cancer survivors should be aware of the close monitoring necessary throughout this period [126]. Healthcare providers need to reassure women that breastfeeding has not proved to increase the risk of relapse. Galactagogues are often phytoestrogens in a concentrated form, which could promote oncogenesis or decrease the efficacy of hormonal therapy [127]. Domperidone and other drugs inducing prolactin release may be contraindicated due to an increased risk of breast cancer development with elevated prolactin [126,128].

## 9. Diabetes Mellitus and Breastfeeding

Preexisting diabetes mellitus (DM) type I or II affects 1–1.5% of all pregnancies and may lead to adverse maternal and neonatal outcomes [129]. Breastfeeding rates among women with preexisting DM are very low; relevant studies mainly include women with DM type I [129,130,131]. Both exclusive and any breastfeeding rates are lower in women with type I DM vs. non-diabetic mothers [132]. Herskin et al. [133] reported different rates of breastfeeding between women with DM type I and type II, both at hospital discharge (76% vs. 45%, respectively) and at 4 months after labor (49% vs. 23%, respectively). Decreased prevalence of breastfeeding may be due to increased morbidity, hospital practices that do not support exclusive breastfeeding, and issues with glycemic control of the mother. Initiation of breastfeeding is frequently challenging for women with DM because of high rates of pregnancy and labor complications, cesarean section and neonatal morbidity, including prematurity, respiratory distress, IUGR, and congenital anomalies [134,135,136]. Neonatal hypoglycemia could also affect the mode of feeding. Hypoglycemia is related to intrauterine hyperglycemia and subsequent fetal hyperinsulinism [137]. Moreover, early mother–child separation can hinder breastfeeding [138]. Establishment of stage II of lactogenesis is delayed by 24 h in mothers with DM type I; however, the milk production at 7 days after labor is similar to that of non-diabetic mothers [139]. Neonates of mothers with type I DM exhibit more immature breastfeeding reflex [140]. Early breastfeeding initiation could reduce neonatal borderline hypoglycemia and increase mean plasma glucose levels [141]. Maternal diet and insulin dosing should be closely monitored due to the postpartum change in glucose levels to ensure adequate control. Fluctuation of plasma glucose levels can delay production of breastmilk and lead to poor supply. Milk production depends on the proper development of breast during pregnancy. Insulin metabolism controls milk secretion, supporting mammary gland differentiation [142]. Insulin upregulates genes related to mammary epithelial cell (MEC) proliferation and downregulates genes responsible for MEC differentiation [143,144]. Insulin resistance may be associated with decreased secretory differentiation and subsequent reduced milk production [145]. The need for insulin of an exclusively breastfeeding mother is often reduced in up to 50% of pre-pregnancy requirements [146]. For infants of mothers with type I or II DM, breastfeeding possibly bears greater advantages. Breastfeeding protects against infectious diseases contributing to the infant’s short-term immunity. Lately, a long-term protective effect of breastfeeding against obesity and type II DM has been confirmed [147,148,149,150]. Longer duration of breastfeeding correlated with lower incidence of type II DM in several studies globally [148,149,150]. Breastfeeding is estimated to reduce the risk of type II DM by up to 50% [151].

## 10. Thyroid Disorders and Breastfeeding

### 10.1. Hypothyroidism

Maternal hypothyroidism is not a contraindication for breastfeeding. Treatment with thyroxine continues throughout lactation. Thyroid hormones are essential for the normal development of the mammary gland and initiation of lactation. T4 and T3 levels are significantly correlated with milk production [152]. Thyroid insufficiency negatively impacts breastmilk supply [153]. In a study in rats, oxytocin levels and milk production were lower in breastfeeding mothers with hypothyroidism, while triglyceride concentration was also reduced in their milk [154].

### 10.2. Hyperthyroidism

During pregnancy and lactation, hyperthyroidism is usually managed with anti-thyroid drugs, which inhibit the synthesis of thyroid hormones. Treatment with radioactive iodine and surgical removal of the thyroid gland are reserved for cases refractory to medicines. Hyperthyroidism is not a contraindication for breastfeeding, but the drugs administered to the mother should be taken into consideration [155]. Based on the literature, both propylthiouracil and methimazole in moderate doses (<300 mg/day for propylthiouracil and 20–30 mg/day for methimazole) are safe during lactation, as their concentration in breastmilk is minimal [156]. The drugs are preferably taken in divided doses, immediately after breastfeeding, to avoid the period of maximum plasma drug levels [155,157]. Close clinical and laboratory monitoring of the mother and the child are necessary. Breastfeeding is contraindicated during treatment with radioactive iodine and for at least 4 weeks after cessation of the therapy [156]. 

### 10.3. Postpartum Thyroiditis

Postpartum thyroiditis is an autoimmune thyroid condition, presenting within the first year after labor, in women without clinical evidence of thyroid dysfunction before pregnancy. Its incidence is estimated at approximately 7% of women and varies depending on genetic background and iodine intake [158]. Women with type I DM are at increased risk of postpartum thyroiditis. The hyperthyroidic period presents 2–6 months after labor, often at 3 months, with 1/3 of affected women being asymptomatic. Otherwise, they could have issues with the care of the infant due to anxiety and nervousness [158]. Lactation is usually unaffected. The hypothyroidic period presents 3–12 months after labor, often at 8 months, with 20–64% of cases leading to permanent hypothyroidism. Symptoms like tiredness, loss of concentration, memory problems, constipation, lack of interest in the infant’s care, and depression can easily be confused for postpartum depression in women who have recently given birth [159,160].

Traces of maternal thyroid hormones are excreted in breastmilk. Thyroxin concentration was measured at 0.83 μg/L, which does not have a significant impact on the status of infantile thyroid hormones [156]. In cases of other autoimmune diseases, hyperprolactinemia of lactation has been considered as a risk factor for disease exacerbation [161]. There are no data regarding the effect of breastfeeding on the presentation or exacerbation of thyroiditis [156].

## 11. Chronic Infectious Diseases and Breastfeeding

### 11.1. Human Immunodeficiency Virus (HIV) Infection and Breastfeeding

During the last decades, significant progress concerning mother-to-child transmission of HIV has been observed [162]. The virus is excreted in breastmilk and can be transmitted during lactation. Viral, maternal, and infant factors affect the risk of transmission. The viral load in the breastmilk is a determinant factor. Lifelong antiretroviral therapy is currently the gold standard for prevention of mother-to-child transmission in case of breastfeeding. In addition to maternal antiretroviral treatment, neonatal post-exposure neonatal prophylaxis is recommended. Initiation of antiretroviral therapy prior to pregnancy is optimal. If sustainably undetectable viral load has been achieved, the risk of transmission is estimated to be up to 1%. Elimination of this risk requires formula feeding and non-breastfeeding. In low- and middle-income countries, WHO recommends exclusive breastfeeding in HIV-infected adherent to therapy women for six months followed by complementary introduction of solid foods and continuation of breastfeeding for two years or longer [163]. In high-resources settings, most organizations encourage patient-centered, evidence-based counselling on infant feeding options [164]. 

Mothers with HIV should be offered constant access to antiretroviral treatment and continuous support in their decision to breastfeed. Close viral monitoring is necessary in lactating mothers to promptly identify any rise of viral load and appropriately modify treatment. Antiretroviral treatment is considered safe during pregnancy and lactation, as their excretion in breastmilk is negligible [165]. 

Women with HIV face additional barriers for exclusive breastfeeding, including disease-related stigma and the burden of a chronic condition, compared to HIV-uninfected mothers [166]. Exclusive breastfeeding is proposed by WHO as a strategy preventing mother-to-child transmission of HIV, adding to the other well-known benefits of breastfeeding. Results from a study in Kenya indicated that rates of breastfeeding initiation and exclusive breastfeeding at six months were higher among HIV-infected women compared to those of non-infected mothers [166]. 

### 11.2. Human T-Cell Lymphotropic Virus Type-I (HTLV-I) Infection and Breastfeeding

Breastfeeding has been reported to be the main source of vertical transmission of vertical transmission of HTLV-I accounted for approximately 95% of mother-to-child transmission cases [167,168]. A meta-analysis conducted by Boostani et al. [167] showed that a short period (less than 6 months) of breastfeeding does not increase the likelihood of mother-to-child transmission of HTLV-I infection, while breastfeeding of longer than 6 months greatly increases the rate of HTLV-I transmission. A recent meta-analysis [168] showed that there was no significant increase in the risk of mother-to-child transmission when breast-feeding lasted for ≤3 months compared with exclusively formula-fed infants (pooled relative risk (RR), 0.72; 95% confidence interval (CI), 0.30–1.77), but there was an almost threefold increase in risk when breastfeeding was carried out for up to 6 months (RR, 2.91, 95% CI, 1.69–5.03).

### 11.3. Hepatitis B Virus (HBV)-Infected Mothers and Breastfeeding

Vertical transmission of HBV is a prevalent cause of HBV spread, resulting in an estimated 50% of the global chronic infections [169]. Routine screening of pregnant women and universal active and passive immunization of neonates have decreased mother-to-child transmission by 95%. HBV has been detected in breastmilk and concerns for transmission through lactation have been raised and investigated. Numerous studies, even before the introduction of HBV vaccine, have not confirmed an increase in the risk of mother-to-child transmission. Therefore, WHO recommends breastfeeding in case of maternal chronic hepatitis B irrespective of the mother’s disease status and availability of the vaccine [170]. A 2011 meta-analysis reported that despite infectiousness of breastmilk, breastfeeding is not associated with increased risk of infantile HBV infection [171]. This finding was consistent even in mothers with high infectivity, probably due to vaccine protection and transmission during delivery.

Antiviral treatment is recommended during pregnancy in selected cases of chronic HBV with high viral load in an attempt to minimize transmission during gestation and delivery [165]. Treatment may need to continue postnatally, during lactation. Nucleoside analogues are recommended during pregnancy; however, due to paucity of published studies, their safety during breastfeeding is not established [172]. It has been confirmed that fetuses in utero are exposed to significantly higher drug levels than infants through breastmilk. Additionally, the same medicines are recommended for use in lactating mothers with HIV. Cumulating evidence suggests that mothers with chronic HBV on antiviral therapy should be encouraged to breastfeed.

### 11.4. Hepatitis C Virus (HCV)-Infected Mothers and Breastfeeding

Infection with HCV remains a public health concern and its incidence among women of child-bearing age warrants screening during pregnancy. The risk of vertical transmission of HCV is estimated at 6%, rising to 10% in case of maternal HIV co-infection [173]. Although HCV is detected in breastmilk, avoidance of breastfeeding does not seem to reduce the risk of mother-to-child transmission [174]. Breastfeeding is recommended unless bleeding or cracked nipples are present. Nevertheless, HCV-infected women breastfeed at lower rates compared to the general population. Mothers with chronic HCV infection should be informed and supported during lactation.

Safety of direct-acting antiviral medicine used for the treatment of HCV has not been studied in lactating women [175]. Data from animal studies indicate that the drugs are excreted into breastmilk but have not demonstrated adverse effects on the offspring. Currently, until published evidence becomes available, these medicines are not recommended for use during pregnancy and lactation. 

## 12. Discussion

Cumulative evidence suggests that women with chronic diseases present lower breastfeeding rates compared with healthy women. Both exclusive and any breastfeeding duration for women with preexisting DM is reduced compared to those of non-diabetic mothers [132,176,177]. Furthermore, women with polycystic ovary syndrome (PCOS) [178], IBD [64], arthritis [55], and epilepsy [78,79] breastfeed less often than non-affected women. Adjusted and evidence-based support may be required to promote breastfeeding in women with chronic diseases; however, our comprehension of breastfeeding in this subpopulation is still unclear. The literature related to breastfeeding extends in various scientific areas and multidisciplinary effort is necessary to compile an overview of current evidence and knowledge regarding breastfeeding issues in mothers with chronic diseases. 

Scime et al. [179], in a cross-sectional study using data from the 2015/2016 Canadian Community Health Survey (CCHS), assessed the probable correlation between breastfeeding and chronic maternal diseases after adjustment for possible socio-demographic confounding factors. The prevalence of self-reported chronic diseases was 11.9% (95% CI 9.8–14.1); musculoskeletal problems and hypertension were the most common conditions. Another study, conducted in the USA in 2008 reported 26.6% of pregnant women suffering from a chronic physical or psychological disease, most frequently arthritis (6.3%), hypertension (5.7%) and asthma (5.0%) [180]. A recent population study in Denmark exhibited a 15.8% of pregnant women with chronic diseases [21]. Arterial hypertension remains the main cause of non-infectious morbidity in the general population [181], which is also reflected during pregnancy. 

Women with chronic diseases seem to begin to breastfeed independently of the disease status. This can be explained by the high intrahospital postpartum support of breastfeeding. No correlation was found between pre-existing diabetes and initiation of breastfeeding (adjusted odds ratio (aOR) 0.9, 95% CI 0.9–1.0) after adjusting for maternal and obstetric complications [182] in a study from Ohio. Moffatt et al. [63] also reported that 83.3% of IBD patients vs. 77.1% of the general population began breastfeeding (*p* > 0.05). No relationship between PCOS and initiation of breastfeeding was recorded (aOR 0.9, 95% CI 0.6–1.4) [183]. Similarly, Scime et al. [179] highlighted comparable non-breastfeeding rates in women with (10.4%) and without (8.7%) chronic disease after adjustment for confounding factors (aOR 0.96, 95% CI 0.54–1.71). Women with chronic disease presented similar rates of early (<6 months) cessation of any breastfeeding (aOR 1.40, 95% CI 0.82–2.40), but a more than twofold risk of early cessation of exclusive breastfeeding (aOR 2.48, 95% CI 1.49–4.12) compared to healthy women. A Swedish prospective cohort study resulted in a significantly lower percentage of any breastfeeding at 6 months in women with type I DM compared to non-diabetic mothers (61.5% vs. 76.7%, respectively, *p* = 0.025), but no difference in exclusive breastfeeding at 6 months (44.4% vs. 40.5%, respectively, *p* = 0.729) [136]. Comparable rates of any breastfeeding for more than 24 weeks were also reported between women with and without IBD (56.1% vs. 44.4%, respectively, *p* = 0.02) [63].

The protective effect of breastfeeding is more powerful in case of exclusive nutrition with maternal milk and is reduced with other supplemental feeding [184]. Findings of various studies indicate that the presence of a chronic maternal disease may negatively impact exclusive, but not partial breastfeeding rates at 6 months [57,179]. A probable explanation for the lower exclusive breastfeeding percentages in mothers with chronic diseases lies in the possible delay of stage II lactogenesis due to the condition itself or the treatment [185,186]. Women with delayed milk production (more than 72 h after birth) are faced with a higher risk of early exclusive breastfeeding cessation compared to mothers without delayed milk production [187]. Labor via cesarean section or need for NICU admission of the neonate are more common among women with chronic diseases and could inhibit attempts to exclusive breastfeeding because of the early (or prolonged) mother-child separation and the maternal stress [188,189]. Disease activity may be dependent on the changes induced by lactation, including the fluctuations (as in endocrine conditions [146]) or recess (as in autoimmune conditions [63,74]) of symptoms, and could affect decisions on breastfeeding. Although very few drugs are contraindicated during breastfeeding, women often receive discouraging advice concerning safety of medicines in lactation [190,191]. Finally, mothers’ desire to “regain a sense of physical fitness”, tiredness and exhaustion from neonatal care, and the feeling of physical or psychological unrest can also contribute to breastfeeding cessation [192]. Mothers with chronic diseases are sensitive to the gift of health and would choose the optimal feeding mode with short-term and long-term benefits for their offspring at the beginning of their life. 

## 13. Conclusions

In-depth knowledge of the pathology of systematic diseases and the characteristics of the recommended treatments is vital when lactation is considered. Mothers with chronic diseases should be offered the possibility to breastfeed their infants, along with the necessary information, education, and support for this endeavor.

In case the therapeutic approach of the mother requires a drug possibly harmful for the infant, the choice between the treatment and breastfeeding is imminent. The mother, together with the healthcare professionals, needs to weigh the advantages of breastfeeding over the drug for the maternal and neonatal health and relationship. Recent technological advancements have helped analyze human milk, allowing for a better understanding of the complex protective mechanisms with long-term consequences. Maternal decisions on breastfeeding are affected by multiple physiological, obstetric, and psychological factors. Further research is necessary for a more effective comprehension of these determinants and the optimal support of breastfeeding in mothers with chronic diseases. Studies indicate that mothers with chronic conditions may benefit from the appropriate care and support in the hospital and in the society that can contribute to the establishment and maintenance of exclusive breastfeeding. 

## Data Availability

Data are contained within the article.

## Supplementary Materials

The following supporting information can be downloaded at: https://www.mdpi.com/article/10.3390/nu15132822/s1, Table S1: Characteristics of retrieved studies.
