# Supplementary material for: Knowledge Gaps and Current Evidence Regarding Breastfeeding Issues in Mothers with Chronic Diseases
Source: Nutrients. 2023 Jun 21;15(13):2822. doi: 10.3390/nu15132822 (PMC10343612; doi:10.3390/nu15132822)
Supplement: Supplementary file 1 [file nutrients-15-02822-s001.zip › nutrients-2442058-supplementary.pdf]

Table S1. Characteristics of retrieved studies.

Country	Country	Design	Sample Sizea	Study outcome
Systematic lupus erythematosus (SLE)				
Chen 2020 [1]	Taiwan	Case-control	10295 women	An increased risk of maternal complications and adverse birth outcomes was observed for SLE patients.
Bundhun 2017 [2]	China	Meta-analysis	529,778 women	SLE had a high impact on maternal and fetal adverse outcomes following pregnancy.
Orefice 2021[3]	Italy	Observational	57 pregnancies in 43 SLE women	The vast majority of mothers with SLE (96.5%) were planning to breastfeed during pregnancy and 71.9% did breastfeed. A relationship between treatment with hydroxychloroquine (HCQ) and delayed breastfeeding cessation was reported for the first time.
Noviani 2016 [4]	USA	Prospective cohort	84 women	A total of 49% of participating SLE patients decided to breastfeed. This rate was not significantly affected by socioeconomic factors. Furthermore, disease activity after labor, full-term labor, breastfeeding education and planning were positively correlated with breastfeeding.
Acevedo 2017 [5]	Argentina	Cross-sectional	62 women	Reduced breastfeeding rates and duration were observed in SLE patients. The initiation of a new treatment was the main reason for breastfeeding cessation in spite of the fact that these drugs were low risk for breastfeeding.
Clowse 2022 [6]		Meta-analysis	7 studies	Current recommendations advocate for the initiation of HCQ when pregnancy is scheduled and the continuation of the drug throughout pregnancy and lactation.
Arthritis (RA)				
Silman 1992 [7]	England	Case-control	232 women	Risk for RA onset increased during the postpartum period, particularly after the first pregnancy.
Brennan 1994 [8]	UK	Case-control	336	The women who breastfed after the first pregnancy had a significantly increased risk for RA development. The risk for RA development was less increased following breastfeeding in a second and not increased in a third pregnancy.
Barrett 2000 [9]	UK	Prospective cohort	137 women	Following adjustment for possible confounding factors including treatment, patients who were breastfeeding for the first time showed increased disease activity 6 months after labor, indicated by self-reported symptoms, number of affected joints, and C-reactive protein levels.
Eudy 2017 [10]	USA	Cross-sectional	150 women	Most women with RA breastfed and were regularly receiving treatment during lactation. Disease activity seemed to worsen, in particular for the patients who did not receive treatment during lactation, while improvement was only observed in women who followed treatment during breastfeeding.

Ince-Askan 2019 [11]	Netherlands	Prospective cohort	3,225 women	Only 4% of mothers with arthritis exclusively breastfed until 26 weeks compared to 25% of the general population.
Inflammatory bowel disease (ibd)				
Nielsen 1984 [12]	N/A	Comparative study	109 pregnancies in 68 women	Pregnancy was not associated with an increased risk of Crohn's disease flare-ups.
Mahadevan 2007 [13]	USA	Cohort study	954 women	Women with IBD were at increased risk for spontaneous abortions, preterm labor, and low birth weight neonates.
Dotan 2013 [14]	Israel	Prospective cohort	334 women	Approximately 1/3 of mothers with IBD did not breastfeed at all compared to 1/5 of healthy controls ($p < 0.005$), and short-term and long-term breastfeeding were also less common in mothers with IBD. Moreover, mothers who received treatment with immunomodulators and steroids had significantly lower breastfeeding rates in comparison to women who were only administered 5-ASA.
Kane 2005 [15]	USA	Cross-sectional	122 women	A significant number of IBD patients did not breastfeed. A relationship between breastfeeding and disease activity may be owing to IBD treatment cessation.
Moffatt 2009 [16]	Canada	Cross-sectional	132 women	IBD did not seem to reduce chances of breastfeeding. Lactation is not associated with increased risk of flares; contrarily, it could be protective during the first year after labor.
Koletzko 1999 [17]	Canada	Questionnaire based	145 families/ 128 children	Breastfeeding was associated with prevention of IBD in children.
Orholm 1999 [18]	Denmark	Cross-sectional	145 women	An increased risk for ulcerative colitis and Crohn's disease was observed in offspring of mothers with IBD compared with the background population [19].
Multiple sclerosis (MS)				
Krysko 2019 [20]	USA	Systematic Review and Meta-analysis	24 studies	Breastfeeding was correlated with lower rate of postpartum MS relapses, with this beneficial effect being greater in cases of increased disease activity and exclusive breastfeeding.
Hellwig 2015 [19]	Germany	Prospective cohort	201 women	Exclusive breastfeeding should be considered as a modestly effective MS treatment.
Jesus-Ribeiro 2017 [21]	Portugal	Retrospective cohort	97 women	Breastfeeding had no impact on the presence of postpartum MS relapses.
Langer-Gould 2020 [22]	USA	Prospective cohort	375 women	Exclusive breastfeeding reduced the risk of early postpartum relapses.
Pakpoor 2013 [23]	N/A	Review	12 studies	Non-breastfeeding women had double the risk of postpartum relapse compared to breastfeeding mothers.
Portaccio 2013 [24]	Italy	Prospective cohort	350 pregnancies	In women with higher disease activity before and during pregnancy, an increased risk of postpartum relapses and deterioration of existing disability was shown.

VUKusic 2004 [25]	Ireland	Prospective cohort	227 women	MS relapse rates were decreased during the last trimester of pregnancy, but they rose during the first 3 months after labor, with up to 30% of patients relapsing [57]. Postpartum relapses are associated with a high risk of disability.
Epilepsy				
Birnbaum 2019 [26]	USA	Prospective cohort	351 women/345 infants	AED can be found in maternal milk; however, this exposure was limited compared to the transfer through placenta during pregnancy.
Noe 2020 [27]	N/A	Commentary		Women with epilepsy often face unique problems and practical issues with pregnancy and lactation and their breastfeeding rate tends to be low. Most AED are considered safe during lactation and mothers with epilepsy should be encouraged to breastfeed.
Meador 2010 [28]	USA, UK	Prospective cohort	194 women	Women with epilepsy often face unique problems and practical issues with pregnancy and lactation and their breastfeeding rate tends to be low. The cognitive outcomes or breastfed children did not differ from those of non-breastfed children for all antiepileptic drugs (AED) combined.
Meador 2014 [29]	USA, UK	Prospective cohort	177 women/181 children	Breastfed children whose mothers received lamotrigine, carbamazepine, valproic acid, or phenytoin during pregnancy had higher intelligence quotient at 6 years compared to non-breastfed children.
Veiby 2013 [30]	Norway	Prospective cohort	78,744 children	There were no harmful effects of antiepileptic drug exposure through breastfeeding on psychomotor development of children.
Asthma				
Popa 2020 [31]	Romania	Systematic review		Women with asthma were at increased risk of preterm labor, cesarean section, premature rupture of membranes, chorioamnionitis, placenta abruption, low birth weight neonate (the more compromised the pulmonary function, the lower the birth weight), extended hospital stay, and perinatal mortality.
Gluck 2006 [32]	USA	Review		Asthma can negatively affect pregnancy outcome and pregnancy may modify the clinical status of an asthmatic woman. Inadequately treated asthma may cause maternal hypoxemia, which can complicate pregnancy and labor outcome.
Harvey 2020 [33]	Australia	Prospective cohort	605 children	Breastfeeding for more than 6 months was associated with lower risk for bronchiolitis and healthcare utilization in high-risk infants due to maternal asthma, both at 6 and at 12 months.
Wright 2001 [34]	USA	Prospective longitudinal	1246 newborn infants	Breastfeeding was correlated with lower risk of recurrent wheezing during the first 2 years of life, but higher risk of asthma and recurrent wheezing after the age of 6 years only for atopic children of asthmatic mothers.

Guilbert 2007 [35]	USA	Prospective cohort	679 children	In the general pediatric population, longer duration of breastfeeding favorably influenced lung function, but in the presence of maternal asthma, longer breastfeeding was associated with decreased airflows.
Meghan 2017 [36]	Canada	Retrospective cohort	2773 infants	An inverse correlation between breastfeeding of asthmatic mothers and infant wheezing, independent of maternal smoking, education, and other risk factors was determined.
Dixon 2017 [37]	Australia	Prospective cohort	75 women	Differences in concentrations and activation of leUKocytes and cytokines were described in the milk of mothers with and those without asthma.
Jensen 2022 [38]	Australia	Prospective cohort	429 women	Data demonstrated a lower breastfeeding initiation and duration and decreased rates of exclusive breastfeeding in women with asthma compared to those in the general population.
Davies 2020 [39]	UK	Cohort study	251629 infants	Breastfeeding rates tended to be lower in case of drug-treated asthma or prescription discontinuation during pregnancy or postpartum.
Congenital heart disease (CHD)				
Hilfiker-Kleiner 2007 [40]		Observational	16 women	Lactating hormones such as prolactin were associated with aggravation of maternal cardiac diseases and especially postpartum cardiomyopathy (PPCM).
Matsuzaka 2021 [41]	Japan	Retrospective study	153 women	Women with CHD tended to opt for formula feeding from the first month postpartum. No significant differences in postpartum cardiovascular events and levels of brain natriuretic peptide (BNP) associated with breastfeeding were observed.
Sickle cell disease (SCD)				
Early 2022 [42]	USA	Cross-sectional	764 455 total deliveries	The risk for obstetric complications and perinatal mortality was higher in pregnant women with SCD.
Early 2023 [43]	USA	Cross-sectional	5 401 899 deliveries	The risk for severe maternal morbidity was higher in people with SCD compared with deliveries among people without SCD.
Ware 2020 [44]	USA	Prospective cohort	16 women	Due to the minimal amount of hydroxyurea transferred through human milk, it was safe for lactating mothers to receive hydroxyurea.
Thalassemia				
Origa 2019 [45]	Italy	Systematic review		Low rates of breastfeeding in women with thalassemia could be explained by the necessity of re-initiation of treatments like chelates that are contraindicated during lactation.
Petrakos 2016 [46]	Greece	Review		Low rates of breastfeeding in women with thalassemia could be explained by the necessity of re-initiation of treatments like chelates that are contraindicated during lactation.
Malignancies				
Bhurosy 2020 [47]	USA	Systematic review		Breastfeeding was challenging among breast cancer survivors. Conservative surgical and irradiation therapy may reduce but not eliminate the ability of the affected breast to produce milk.

Chelysheva 2018 [48]	Russia	Case series	3 women	Breastfeeding without tyrosine kinase inhibitors (TKI) treatment may be safe preferably only in women with chronic myelogenous leUKemia who have durable deep molecular response. The suggestion for TKIs cessation and breastfeeding for a short period of 2–5 days after labor was also acceptable.
Johnson 2020 [49]	ABM Clinical Protocol #34: Breast Cancer and Breastfeeding			Galactagogues are often phytoestrogens in a concentrated form which could promote oncogenesis or decrease the efficacy of hormonal therapy. Domperidone and other drugs inducing prolactin release may be contraindicated due to an increased risk of breast cancer development with elevated prolactin.
Diabetes mellitus (DM)				
Laine 2021 [50]	Finland	Observational cohort	1089 women	Breastfeeding was estimated to reduce the risk of type II diabetes mellitus.
Evers 2004 [51]	Netherlands	Prospective cohort	323 women/324 infants	Increased maternal and perinatal complications in women with type 1 diabetes.
Jensen 2004 [52]	Denmark	Prospective multicenter study	990 women with 1,218 pregnancies	Type 1 diabetic pregnancies were associated with higher rates of severe perinatal complications compared with the background population.
Webster 1995 [53]	Australia	Prospective cohort	37 pregnant women	Establishment of stage II of lactogenesis was delayed by 24 hours in mothers with insulin-dependent diabetes mellitus, but breastfeeding patterns of these women were comparable to those without diabetes.
Chertok 2009 [54]	USA	Prospective pilot study	84 infants	Early breastfeeding initiation could reduce neonatal borderline hypoglycemia and increase mean plasma glucose levels.
Herskin 2016 [55]	Denmark	Prospective cohort	149 women	Different rates of breastfeeding between women with diabetes mellitus type I and type II, both at hospital discharge (76% versus 45%, respectively) and at 4 months after labor (49% versus 23%, respectively), were reported.
Hummel 2007 [56]	Germany	Prospective cohort	1560 children	Neonates of mothers with type I diabetes mellitus exhibited more immature breastfeeding reflex.
Kachoria 2014 [57]	USA	Cross-sectional	803,222 births	No correlation was found between pre-existing diabetes and initiation of breastfeeding (adjusted odds ratio (aOR) 0.9, 95% CI 0.9–1.0) after adjusting for maternal and obstetric complications.
Kaul 2019 [58]	Canada	Retrospective cohort	65,079 women	A long-term protective effect of breastfeeding against obesity and type II diabetes mellitus was confirmed in all groups except LGA children of mothers with diabetes.
Riviello 2009 [59]	Italy	Prospective cohort	18 women	The need for insulin of an exclusively breastfeeding mother was often reduced in up to 50% of pre-pregnancy requirements.

Sorkio 2010 [60]	Europe,b Canada, USA, Australia	Prospective cohort	2,160 infants	Both exclusive and any breastfeeding rates were lower in women with type I DM versus non-diabetic mothers.
Sparud-Lundin 2011 [61]	Sweden	Prospective cohort	212 women	Initiation and the likelihood of long-term breastfeeding was frequently challenging for women with DM because of high rates of pregnancy and labor complications.
Finkelstein 2013 [62]	Canada	Cross-sectional	24,755 women	Women with insulin-treated diabetes were least likely to breastfeed, followed by women with non-insulin-treated diabetes and women with gestational diabetes compered to women with and without diabetes.
Suwaydi 2022 [63]	Australia	Case series	8 women	A greater risk of delayed secretory activation and low milk supply was reported in women with gestational diabetes mellitus.
Thyroid disorders				
Stuebe 2015 [64]	USA	Longitudinal study	52 women	During the first 8 weeks postpartum, the thyroid profiles of exclusively breastfeeding women changed significantly.
Joshi 1993 [65]	India	Prospective cohort	227 women	Thyroid insufficiency negatively impacted breastmilk supply.
Chronic infectious diseases and breastfeeding				
Ehrhardt 2015 [66]	USA	Review		Antiretroviral treatment was considered safe during pregnancy and lactation, as their excretion in breastmilk is negligible.
Benova 2014 [67]		Meta-analysis	109 studies	The risk of vertical transmission of HCV was estimated at 6%, rising to 10% in case of maternal HIV co-infection.
Tuthil 2020 [68]	Kenya	Longitudinal observational cohort	275 women	Rates of breastfeeding initiation and exclusive breastfeeding at six months were higher among HIV-infected women compared to those of non-infected mothers.
Zheng 2011 [69]	China	Meta-analysis	32 studies	Despite infectiousness of breastmilk, breastfeeding was not associated with increased risk of infantile HBV infection.
Li 2021 [70]	China	Retrospective survey	163 women	HBV-infected mothers who stopped NA treatment at late postpartum period or had less knowledge of medication were more likely to use formula feeding than to breastfeed their baby.
Espinosa 2021 [71]	USA	Retrospective cohort study	404 infants	Low rates of breastfeeding or breast milk initiation at hospital discharge were observed in infants exposed to HCV perinatally.
Boostani 2018 [72]	Iran, USA	Meta-analysis	96 studies	A short period (less than 6 months) of breastfeeding did not increase the likelihood of mother-to-child transmission of HTLV-I infection, while breastfeeding longer than 6 months greatly increased the rate of HTLV-I transmission.

Itabashi 2021 [73]	Japan	Meta-analysis		There was no significant increase in the risk of mother-to-child transmission when breastfeeding lasted for ≤3 months compared with exclusively formula-fed infants, but there was an almost threefold increase in risk when breastfeeding was carried out for up to 6 months.
Polycystic ovarian syndrome (PCOS)				
Vanky 2008 [74]	Norway	Prospective cohort	135 women	Women with PCOS breastfed less often than non-affected women.
Chronic disease overall				
Scime 2022 [75]	Canada	Cross-sectional	2100 women	The prevalence of self-reported chronic diseases was 11.9% (95% CI 9.8–14.1); musculoskeletal problems and hypertension were the most common conditions. The study highlighted comparable non-breastfeeding rates in women with and without chronic disease after adjustment for confounding factors.
Chatterjee 2008 [76]	USA	Cross-sectional	6,294 women	A total of 26.6% of pregnant women suffering from a chronic physical or psychological disease, most frequently arthritis (6.3%), hypertension (5.7%) and asthma (5.0%) was reported.
Jølving 2016 [77]	Denmark	Cohort study	1 362 200 childbirths	A prevalence 15.8% of maternal chronic disease during pregnancy was found.

References

1. Chen, Y.J.; Chang, J.C.; Lai, E.L.; Liao, T.L.; Chen, H.H.; Hung, W.T.; Hsieh, T.Y.; Huang, W.N.; Chen, Y.H.; Lin, C.H.; et al. Maternal and perinatal outcomes of pregnancies in systemic lupus erythematosus: A nationwide population-based study. *Seminars in arthritis and rheumatism* **2020**, *50*, 451-457, doi:10.1016/j.semarthrit.2020.01.014.
2. Bundhun, P.K.; Soogund, M.Z.; Huang, F. Impact of systemic lupus erythematosus on maternal and fetal outcomes following pregnancy: A meta-analysis of studies published between years 2001-2016. *Journal of autoimmunity* **2017**, *79*, 17-27, doi:10.1016/j.jaut.2017.02.009.
3. Orefice, V.; Ceccarelli, F.; Pirone, C.; Galoppi, P.; Spinelli, F.R.; Alessandri, C.; Brunelli, R.; Perrone, G.; Conti, F. Breastfeeding in women affected by systemic lupus erythematosus: Rate, duration and associated factors. *Lupus* **2021**, *30*, 913-920, doi:10.1177/0961203321995263.
4. Noviani, M.; Wasserman, S.; Clowse, M.E. Breastfeeding in mothers with systemic lupus erythematosus. *Lupus* **2016**, *25*, 973-979, doi:10.1177/0961203316629555.
5. Acevedo, M.; Pretini, J.; Micelli, M.; Sequeira, G.; Kerzberg, E. Breastfeeding initiation, duration, and reasons for weaning in patients with systemic lupus erythematosus. *Rheumatology International* **2017**, *37*, 1183-1186, doi:10.1007/s00296-017-3750-1.
6. Megan, E.B.C.; Amanda, M.E.; Stephen, B.; Gillian, S.-S.; Andrzej, K.; Rebecca, F.-B.; Dafna, D.G.; Yair, M.; Cecilia, N.; Abir, M.; et al. Hydroxychloroquine in the pregnancies of women with lupus: a meta-analysis of individual participant data. *Lupus Science & Medicine* **2022**, *9*, e000651, doi:10.1136/lupus-2021-000651.
7. Silman, A.; Kay, A.; Brennan, P. Timing of pregnancy in relation to the onset of rheumatoid arthritis. *Arthritis and rheumatism* **1992**, *35*, 152-155, doi:10.1002/art.1780350205.
8. Brennan, P.; Silman, A. Breast-feeding and the onset of rheumatoid arthritis. *Arthritis and rheumatism* **1994**, *37*, 808-813, doi:10.1002/art.1780370605.
9. Barrett, J.H.; Brennan, P.; Fiddler, M.; Silman, A. Breast-feeding and postpartum relapse in women with rheumatoid and inflammatory arthritis. *Arthritis and rheumatism* **2000**, *43*, 1010-1015, doi:10.1002/1529-0131(200005)43:5<1010::aid-anr8>3.0.co;2-o.
10. Eudy, A.M.; Siega-Riz, A.M.; Engel, S.M.; Franceschini, N.; Howard, A.G.; Clowse, M.E.B.; Petri, M. Effect of pregnancy on disease flares in patients with systemic lupus erythematosus. *Annals of the rheumatic diseases* **2018**, *77*, 855-860, doi:10.1136/annrheumdis-2017-212535.
11. Ince-Askan, H.; Hazes, J.M.W.; Dolhain, R. Breastfeeding among Women with Rheumatoid Arthritis Compared with the General Population: Results from a Nationwide Prospective Cohort Study. *The Journal of rheumatology* **2019**, *46*, 1067-1074, doi:10.3899/jrheum.180805.
12. Nielsen, O.H.; Andreasson, B.; Bondesen, S.; Jacobsen, O.; Jarnum, S. Pregnancy in Crohn's disease. *Scandinavian journal of gastroenterology* **1984**, *19*, 724-732.
13. Mahadevan, U.; Sandborn, W.J.; Li, D.K.; Hakimian, S.; Kane, S.; Corley, D.A. Pregnancy outcomes in women with inflammatory bowel disease: a large community-based study from Northern California. *Gastroenterology* **2007**, *133*, 1106-1112, doi:10.1053/j.gastro.2007.07.019.
14. Dotan, I.; Alper, A.; Rachmilewitz, D.; Israeli, E.; Odes, S.; Chermesh, I.; Naftali, T.; Fraser, G.; Shitrit, A.B.-G.; Peles, V.; et al. Maternal inflammatory bowel disease has short and long-term effects on the health of their offspring: A multicenter study in Israel. *Journal of Crohn's and Colitis* **2013**, *7*, 542-550, doi:10.1016/j.crohns.2012.08.012.
15. Kane, S.; Lemieux, N. The Role of Breastfeeding in Postpartum Disease Activity in Women with Inflammatory Bowel Disease. *Official journal of the American College of Gastroenterology | ACG* **2005**, *100*, 102-105.

16. Moffatt, D.C.; Ilnyckyj, A.; Bernstein, C.N. A Population-Based Study of Breastfeeding in Inflammatory Bowel Disease: Initiation, Duration, and Effect on Disease in the Postpartum Period. *Official journal of the American College of Gastroenterology | ACG* **2009**, *104*.
17. Koletzko, S.; Sherman, P.; Corey, M.; Griffiths, A.; Smith, C. Role of infant feeding practices in development of Crohn's disease in childhood. *BMJ: British Medical Journal* **1989**, *298*, 1617.
18. Orholm, M.; Fonager, K.; Sørensen, H.T. Risk of ulcerative colitis and Crohn's disease among offspring of patients with chronic inflammatory bowel disease. *The American journal of gastroenterology* **1999**, *94*, 3236-3238, doi:10.1111/j.1572-0241.1999.01526.x.
19. Hellwig, K.; Rockhoff, M.; Herbstritt, S.; Borisow, N.; Haghikia, A.; Elias-Hamp, B.; Menck, S.; Gold, R.; Langer-Gould, A. Exclusive Breastfeeding and the Effect on Postpartum Multiple Sclerosis Relapses. *JAMA neurology* **2015**, *72*, 1132-1138, doi:10.1001/jamaneurol.2015.1806.
20. Krysko, K.M.; Rutatangwa, A.; Graves, J.; Lazar, A.; Waubant, E. Association Between Breastfeeding and Postpartum Multiple Sclerosis Relapses: A Systematic Review and Meta-analysis. *JAMA neurology* **2020**, *77*, 327-338, doi:10.1001/jamaneurol.2019.4173.
21. Jesus-Ribeiro, J.; Correia, I.; Martins, A.I.; Fonseca, M.; Marques, I.; Batista, S.; Nunes, C.; Macário, C.; Almeida, M.C.; Sousa, L. Pregnancy in Multiple Sclerosis: A Portuguese cohort study. *Multiple sclerosis and related disorders* **2017**, *17*, 63-68, doi:10.1016/j.msard.2017.07.002.
22. Langer-Gould, A.; Smith, J.; Albers, K.; Wu, J.; Kerezsi, E.; McClearnen, K.; Leimpeter, A.; Van Den Eeden, S. Pregnancy-related relapses in a large, contemporary multiple sclerosis cohort: no increased risk in the postpartum period (S6. 007). **2019**.
23. Pakpoor, J.; Disanto, G.; Lacey, M.V.; Hellwig, K.; Giovannoni, G.; Ramagopalan, S.V. Breastfeeding and multiple sclerosis relapses: a meta-analysis. *Journal of neurology* **2012**, *259*, 2246-2248, doi:10.1007/s00415-012-6553-z.
24. Portaccio, E.; Ghezzi, A.; Hakiki, B.; Sturchio, A.; Martinelli, V.; Moiola, L.; Patti, F.; Mancardi, G.L.; Solaro, C.; Tola, M.R.; et al. Postpartum relapses increase the risk of disability progression in multiple sclerosis: the role of disease modifying drugs. *Journal of neurology, neurosurgery, and psychiatry* **2014**, *85*, 845-850, doi:10.1136/jnnp-2013-306054.
25. Vukusic, S.; Hutchinson, M.; Hours, M.; Moreau, T.; Cortinovis-Tourniaire, P.; Adeleine, P.; Confavreux, C. Pregnancy and multiple sclerosis (the PRIMS study): clinical predictors of post-partum relapse. *Brain : a journal of neurology* **2004**, *127*, 1353-1360, doi:10.1093/brain/awh152.
26. Birnbaum, A.K.; Meador, K.J.; Karanam, A.; Brown, C.; May, R.C.; Gerard, E.E.; Gedzelman, E.R.; Penovich, P.E.; Kalayjian, L.A.; Cavitt, J.; et al. Antiepileptic Drug Exposure in Infants of Breastfeeding Mothers With Epilepsy. *JAMA neurology* **2020**, *77*, 441-450, doi:10.1001/jamaneurol.2019.4443.
27. Noe, K. Further Evidence Breastfeeding by Women With Epilepsy Is Safe: Are Mothers Getting the Message? *Epilepsy currents* **2020**, *20*, 141-143, doi:10.1177/1535759720917997.
28. Meador, K.J.; Baker, G.A.; Browning, N.; Clayton-Smith, J.; Combs-Cantrell, D.T.; Cohen, M.; Kalayjian, L.A.; Kanner, A.; Liporace, J.D.; Pennell, P.B.; et al. Effects of breastfeeding in children of women taking antiepileptic drugs. *Neurology* **2010**, *75*, 1954-1960, doi:10.1212/WNL.0b013e3181ffe4a9.
29. Meador, K.J.; Baker, G.A.; Browning, N.; Cohen, M.J.; Bromley, R.L.; Clayton-Smith, J.; Kalayjian, L.A.; Kanner, A.; Liporace, J.D.; Pennell, P.B.; et al. Breastfeeding in children of women taking antiepileptic drugs: cognitive outcomes at age 6 years. *JAMA pediatrics* **2014**, *168*, 729-736, doi:10.1001/jamapediatrics.2014.118.

30. Veiby, G.; Engelsen, B.A.; Gilhus, N.E. Early child development and exposure to antiepileptic drugs prenatally and through breastfeeding: a prospective cohort study on children of women with epilepsy. *JAMA neurology* **2013**, *70*, 1367-1374, doi:10.1001/jamaneurol.2013.4290.
31. Popa, M.; Peltecu, G.; Gica, N.; Ciobanu, A.M.; Botezatu, R.; Gica, C.; Steriade, A.; Panaitescu, A.M. Asthma in Pregnancy. Review of Current Literature and Recommendations. *Maedica* **2021**, *16*, 80-87, doi:10.26574/maedica.2020.16.1.80.
32. Gluck, J.C.; Gluck, P.A. The effect of pregnancy on the course of asthma. *Immunology and allergy clinics of North America* **2006**, *26*, 63-80, doi:10.1016/j.iac.2005.10.008.
33. Harvey, S.M.; Murphy, V.E.; Gibson, P.G.; Collison, A.; Robinson, P.; Sly, P.D.; Mattes, J.; Jensen, M.E. Maternal asthma, breastfeeding, and respiratory outcomes in the first year of life. *Pediatric Pulmonology* **2020**, *55*, 1690-1696, doi:https://doi.org/10.1002/ppul.24756.
34. Wright, A.L.; Holberg, C.J.; Taussig, L.M.; Martinez, F.D. Factors influencing the relation of infant feeding to asthma and recurrent wheeze in childhood. *Thorax* **2001**, *56*, 192-197, doi:10.1136/thorax.56.3.192.
35. Guilbert, T.W.; Stern, D.A.; Morgan, W.J.; Martinez, F.D.; Wright, A.L. Effect of breastfeeding on lung function in childhood and modulation by maternal asthma and atopy. *American journal of respiratory and critical care medicine* **2007**, *176*, 843-848, doi:10.1164/rccm.200610-1507OC.
36. Meghan, B.A.; Lorena, V.; Zihang, L.; David, D.; Padmaja, S.; Allan, B.B.; Piushkumar, J.M.; Stuart , E.T.; Diana, L.L.; Malcolm, R.S. Breastfeeding, maternal asthma and wheezing in the first year of life: a longitudinal birth cohort study. *European Respiratory Journal* **2017**, *49*, 1602019, doi:10.1183/13993003.02019-2016.
37. Dixon, D.L.; Forsyth, K.D. Leukocytes in expressed breast milk of asthmatic mothers. *Allergologia et immunopathologia* **2017**, *45*, 325-332, doi:10.1016/j.aller.2016.08.015.
38. Jensen, M.E.; Harvey, S.M.; Gibson, P.G.; Murphy1, V.E. Breastfeeding rates and barriers in women with asthma. In Proceedings of the 46th Annual Scientific Meeting of the Nutrition Society of Australia, 29 November – 2 December 2022, Sustainable nutrition for a healthy life, 2022.
39. Davies, G.; Jordan, S.; Thayer, D.; Tucker, D.; Humphreys, I. Medicines prescribed for asthma, discontinuation and perinatal outcomes, including breastfeeding: A population cohort analysis. *PLOS ONE* **2020**, *15*, e0242489, doi:10.1371/journal.pone.0242489.
40. Hilfiker-Kleiner, D.; Kaminski, K.; Podewski, E.; Bonda, T.; Schaefer, A.; Sliwa, K.; Forster, O.; Quint, A.; Landmesser, U.; Doerries, C.; et al. A cathepsin D-cleaved 16 kDa form of prolactin mediates postpartum cardiomyopathy. *Cell* **2007**, *128*, 589-600, doi:10.1016/j.cell.2006.12.036.
41. Matsuzaka, Y.; Aoki-Kamiya, C.; Yokouchi-Konishi, T.; Shionoiri, T.; Nakanishi, A.; Iwanaga, N.; Chishaki, A.; Kurosaki, K.; Ohuchi, H.; Yoshimatsu, J. Breastfeeding and postpartum outcomes among women with congenital heart disease. *International Journal of Cardiology Congenital Heart Disease* **2021**, *4*, 100167, doi:https://doi.org/10.1016/j.ijcchd.2021.100167.
42. Early, M.L.; Eke, A.C.; Gemmill, A.; Lanzkron, S.; Pecker, L.H. Comparisons of Severe Maternal Morbidity and Other Adverse Pregnancy Outcomes in Pregnant People With Sickle Cell Disease vs Anemia. *JAMA Network Open* **2023**, *6*, e2254545-e2254545, doi:10.1001/jamanetworkopen.2022.54545.
43. Early, M.L.; Eke, A.C.; Gemmill, A.; Lanzkron, S.; Pecker, L.H. Severe Maternal Morbidity and Mortality in Sickle Cell Disease in the National Inpatient Sample, 2012-2018. *JAMA Network Open* **2023**, *6*, e2254552-e2254552, doi:10.1001/jamanetworkopen.2022.54552.

44. Ware, R.E.; Marahatta, A.; Ware, J.L.; McElhinney, K.; Dong, M.; Vinks, A.A. Hydroxyurea Exposure in Lactation: a Pharmacokinetics Study (HELPS). *The Journal of pediatrics* **2020**, *222*, 236-239, doi:https://doi.org/10.1016/j.jpeds.2020.02.002.
45. Origa, R.; Comitini, F. Pregnancy in Thalassemia. *Mediterranean journal of hematology and infectious diseases* **2019**, *11*, e2019019, doi:10.4084/mjhid.2019.019.
46. Petrakos G, A.P., Tsironi M. Pregnancy in women with thalassemia: challenges and solutions. *Int J Womens Health.* **2016**, *8*, 441-451, doi:https://doi.org/10.2147/IJWH.S89308.
47. Bhurosy, T.; Niu, Z.; Heckman, C.J. Breastfeeding is Possible: A Systematic Review on the Feasibility and Challenges of Breastfeeding Among Breast Cancer Survivors of Reproductive Age. *Annals of surgical oncology* **2021**, *28*, 3723-3735, doi:10.1245/s10434-020-09094-1.
48. Chelysheva, E.; Aleshin, S.; Polushkina, E.; Shmakov, R.; Shokhin, I.; Chilov, G.; Turkina, A. Breastfeeding in Patients with Chronic Myeloid Leukaemia: Case Series with Measurements of Drug Concentrations in Maternal Milk and Literature Review. *Mediterranean journal of hematology and infectious diseases* **2018**, *10*, e2018027, doi:10.4084/mjhid.2018.027.
49. Johnson, H.M.; Mitchell, K.B. ABM Clinical Protocol #34: Breast Cancer and Breastfeeding. *Breastfeeding medicine : the official journal of the Academy of Breastfeeding Medicine* **2020**, *15*, 429-434, doi:10.1089/bfm.2020.29157.hmj.
50. Laine, M.K.; Kautiainen, H.; Gissler, M.; Pennanen, P.; Eriksson, J.G. Impact of gestational diabetes mellitus on the duration of breastfeeding in primiparous women: an observational cohort study. *International Breastfeeding Journal* **2021**, *16*, 19, doi:10.1186/s13006-021-00369-1.
51. Evers, I.M.; de Valk, H.W.; Visser, G.H. Risk of complications of pregnancy in women with type 1 diabetes: nationwide prospective study in the Netherlands. *BMJ (Clinical research ed.)* **2004**, *328*, 915, doi:10.1136/bmj.38043.583160.EE.
52. Jensen, D.M.; Damm, P.; Moelsted-Pedersen, L.; Ovesen, P.; Westergaard, J.G.; Moeller, M.; Beck-Nielsen, H. Outcomes in type 1 diabetic pregnancies: a nationwide, population-based study. *Diabetes care* **2004**, *27*, 2819-2823, doi:10.2337/diacare.27.12.2819.
53. Webster, J.; Moore, K.; McMullan, A. Breastfeeding outcomes for women with insulin dependent diabetes. *Journal of human lactation : official journal of International Lactation Consultant Association* **1995**, *11*, 195-200, doi:10.1177/089033449501100320.
54. Chertok, I.R.; Raz, I.; Shoham, I.; Haddad, H.; Wiznitzer, A. Effects of early breastfeeding on neonatal glucose levels of term infants born to women with gestational diabetes. *Journal of human nutrition and dietetics : the official journal of the British Dietetic Association* **2009**, *22*, 166-169, doi:10.1111/j.1365-277X.2008.00921.x.
55. Herskin, C.W.; Stage, E.; Barfred, C.; Emmersen, P.; Ladefoged Nichum, V.; Damm, P.; Mathiesen, E.R. Low prevalence of long-term breastfeeding among women with type 2 diabetes. *The journal of maternal-fetal & neonatal medicine : the official journal of the European Association of Perinatal Medicine, the Federation of Asia and Oceania Perinatal Societies, the International Society of Perinatal Obstet* **2016**, *29*, 2513-2518, doi:10.3109/14767058.2015.1092138.
56. Hummel, S.; Winkler, C.; Schoen, S.; Knopff, A.; Marienfeld, S.; Bonifacio, E.; Ziegler, A.G. Breastfeeding habits in families with Type 1 diabetes. *Diabetic medicine : a journal of the British Diabetic Association* **2007**, *24*, 671-676, doi:10.1111/j.1464-5491.2007.02148.x.
57. Kachoria, R.; Oza-Frank, R. Differences in breastfeeding initiation by maternal diabetes status and race, Ohio 2006-2011. *Maternal and child health journal* **2014**, *18*, 2226-2235, doi:10.1007/s10995-014-1472-5.
58. Kaul, P.; Bowker, S.L.; Savu, A.; Yeung, R.O.; Donovan, L.E.; Ryan, E.A. Association between maternal diabetes, being large for gestational age and breast-feeding on being overweight or obese in childhood. *Diabetologia* **2019**, *62*, 249-258, doi:10.1007/s00125-018-4758-0.

59. Riviello, C.; Mello, G.; Jovanovic, L.G. Breastfeeding and the basal insulin requirement in type 1 diabetic women. *Endocrine practice : official journal of the American College of Endocrinology and the American Association of Clinical Endocrinologists* **2009**, *15*, 187-193, doi:10.4158/ep.15.3.187.
60. Sorkio, S.; Cuthbertson, D.; Bärlund, S.; Reunanen, A.; Nucci, A.M.; Berseth, C.L.; Koski, K.; Ormisson, A.; Savilahti, E.; Uusitalo, U.; et al. Breastfeeding patterns of mothers with type 1 diabetes: results from an infant feeding trial. *Diabetes/metabolism research and reviews* **2010**, *26*, 206-211, doi:10.1002/dmrr.1074.
61. Sparud-Lundin, C.; Wennergren, M.; Elfvin, A.; Berg, M. Breastfeeding in women with type 1 diabetes: exploration of predictive factors. *Diabetes care* **2011**, *34*, 296-301, doi:10.2337/dc10-1916.
62. Finkelstein, S.A.; Keely, E.; Feig, D.S.; Tu, X.; Yasseen, A.S., 3rd; Walker, M. Breastfeeding in women with diabetes: lower rates despite greater rewards. A population-based study. *Diabetic medicine : a journal of the British Diabetic Association* **2013**, *30*, 1094-1101, doi:10.1111/dme.12238.
63. Suwaydi, M.A.; Wlodek, M.E.; Lai, C.T.; Prosser, S.A.; Geddes, D.T.; Perrella, S.L. Delayed secretory activation and low milk production in women with gestational diabetes: a case series. *BMC Pregnancy and Childbirth* **2022**, *22*, 350, doi:10.1186/s12884-022-04685-0.
64. Stuebe, A.M.; Meltzer-Brody, S.; Pearson, B.; Pedersen, C.; Grewen, K. Maternal neuroendocrine serum levels in exclusively breastfeeding mothers. *Breastfeeding medicine : the official journal of the Academy of Breastfeeding Medicine* **2015**, *10*, 197-202, doi:10.1089/bfm.2014.0164.
65. Joshi, J.V.; Bhandarkar, S.D.; Chadha, M.; Balaiah, D.; Shah, R. Menstrual irregularities and lactation failure may precede thyroid dysfunction or goitre. *Journal of postgraduate medicine* **1993**, *39*, 137-141.
66. Ehrhardt, S.; Xie, C.; Guo, N.; Nelson, K.; Thio, C.L. Breastfeeding while taking lamivudine or tenofovir disoproxil fumarate: a review of the evidence. *Clinical infectious diseases : an official publication of the Infectious Diseases Society of America* **2015**, *60*, 275-278, doi:10.1093/cid/ciu798.
67. Benova, L.; Mohamoud, Y.A.; Calvert, C.; Abu-Raddad, L.J. Vertical transmission of hepatitis C virus: systematic review and meta-analysis. *Clinical infectious diseases : an official publication of the Infectious Diseases Society of America* **2014**, *59*, 765-773, doi:10.1093/cid/ciu447.
68. Tuthill, E.L.; Miller, J.D.; Collins, S.M.; Widen, E.M.; Onono, M.; Young, S.L. HIV infection, hunger, breastfeeding self-efficacy, and depressive symptoms are associated with exclusive breastfeeding to six months among women in western Kenya: a longitudinal observational study. *International Breastfeeding Journal* **2020**, *15*, 4, doi:10.1186/s13006-019-0251-8.
69. Zheng, Y.; Lu, Y.; Ye, Q.; Xia, Y.; Zhou, Y.; Yao, Q.; Wei, S. Should chronic hepatitis B mothers breastfeed? a meta analysis. *BMC Public Health* **2011**, *11*, 502, doi:10.1186/1471-2458-11-502.
70. Li, E.-M.; Xiao, L.-X.; Xu, Z.; Mo, Z.-S.; Li, J.-Q.; Mei, Y.-Y.; Lin, C.-S. Factors associated with non-compliance with breastfeeding recommendation: a retrospective survey in hepatitis B virus-infected mothers who had taken Nucleos(t)ide analogs during pregnancy. *BMC Pregnancy and Childbirth* **2021**, *21*, 551, doi:10.1186/s12884-021-04020-z.
71. Espinosa, C.M.; Niestrath, R.G. Low Breastfeeding Rates and Complex Barriers to Breastfeeding and Breast Milk Utilization in Hepatitis C Perinatally Exposed Infants. *Pediatrics* **2021**, *147*, 323-324, doi:10.1542/peds.147.3MA4.323.
72. Boostani, R.; Sadeghi, R.; Sabouri, A.; Ghabeli-Juibary, A. Human T-lymphotropic virus type I and breastfeeding; systematic review and meta-analysis of the literature. *Iranian journal of neurology* **2018**, *17*, 174-179.
73. Itabashi, K.; Miyazawa, T. Mother-to-Child Transmission of Human T-Cell Leukemia Virus Type 1: Mechanisms and Nutritional Strategies for Prevention. *Cancers* **2021**, *13*, 4100.

74. Vanky, E.; Isaksen, H.; Moen, M.H.; Carlsen, S.M. Breastfeeding in polycystic ovary syndrome. *Acta Obstet Gynecol Scand* **2008**, *87*, 531-535, doi:10.1080/00016340802007676.
75. Scime, N.V.; Patten, S.B.; Tough, S.C.; Chaput, K.H. Maternal chronic disease and breastfeeding outcomes: a Canadian population-based study. *The Journal of Maternal-Fetal & Neonatal Medicine* **2022**, *35*, 1148-1155, doi:10.1080/14767058.2020.1743664.
76. Chatterjee, S.; Kotelchuck, M.; Sambamoorthi, U. Prevalence of chronic illness in pregnancy, access to care, and health care costs: implications for interconception care. *Women's health issues : official publication of the Jacobs Institute of Women's Health* **2008**, *18*, S107-116, doi:10.1016/j.whi.2008.06.003.
77. Jølving, L.R.; Nielsen, J.; Kesmodel, U.S.; Nielsen, R.G.; Beck-Nielsen, S.S.; Nørgård, B.M. Prevalence of maternal chronic diseases during pregnancy - a nationwide population based study from 1989 to 2013. *Acta Obstet Gynecol Scand* **2016**, *95*, 1295-1304, doi:10.1111/aogs.13007.
